# Supplementary material for: 3D collagen high-throughput screen identifies drugs that induce epithelial polarity and enhance chemotherapy response in colorectal cancer
Source: Commun Biol. 2025 Aug 22;8:1261. doi: 10.1038/s42003-025-08699-0 (PMC12373842; doi:10.1038/s42003-025-08699-0)
Supplement: Supplementary file 2 — Supplemental Material [file 42003_2025_8699_MOESM2_ESM.pdf]

SUPPLEMENTAL MATERIAL

3D collagen high-throughput screen identifies drugs that induce epithelial polarity and enhance chemotherapy response in colorectal cancer

Sarah J. Harmych[1,2], Thomas P. Hasaka[3], Chelsie K. Sievers[1], Seung Woo Kang[2,4,5], Marisol A Ramirez[6,7], Vivian Truong Jones[1,8], Zhiguo Zhao[6], Oleg Kovtun[9], Claudia C. Wahoski[1,10], Qi Liu[6,7], Ken S. Lau[2,4,5,11,12], Robert J. Coffey[1,4], Joshua A. Bauer[3,12,13], and Bhuminder Singh[1,2,4,10,12,*]

[1] Department of Medicine, Vanderbilt University Medical Center, Nashville, TN, USA
[2] Department of Cell and Developmental Biology, Vanderbilt University, Nashville, TN, USA
[3] Vanderbilt Institute of Chemical Biology, High-Throughput Screening Facility, Vanderbilt University, Nashville, TN, USA
[4] Epithelial Biology Center, Vanderbilt University Medical Center, Nashville, TN, USA
[5] Center for Computational Systems Biology, Vanderbilt University, Nashville, TN, USA
[6] Department of Biostatistics, Vanderbilt University Medical Center, Nashville, TN 37232, USA
[7] Center for Quantitative Sciences, Vanderbilt University Medical Center, Nashville, TN, USA
[8] Department of Pharmacology, Vanderbilt University, Nashville, TN, USA
[9] Department of Chemistry, Vanderbilt University, Nashville, TN, USA
[10] Program in Cancer Biology, Vanderbilt University, Nashville, TN, USA
[11] Department of Surgery, Vanderbilt University Medical Center, Nashville, TN, USA
[12] Vanderbilt-Cancer Ingram Center, Vanderbilt University Medical Center, Nashville, TN, USA
[13] Department of Biochemistry, Vanderbilt University School of Medicine, Nashville, TN, USA

[*]Corresponding Author:
Bhuminder Singh, PhD
2213 Garland Avenue
10465J, MRB IV
Vanderbilt University Medical Center
E-mail: bhuminder.singh@vumc.org
Nashville, TN 37232-0441

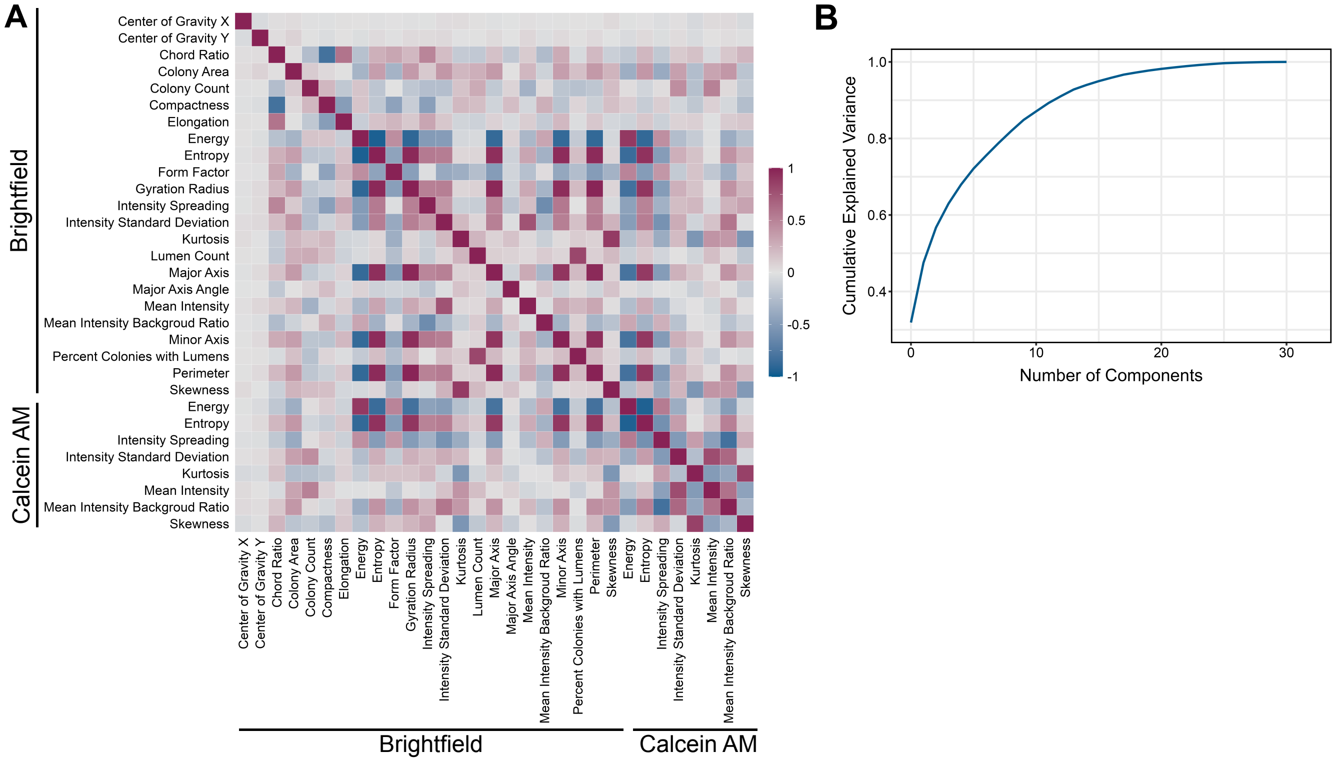

Supplemental Figure 1. Correlation among all the screening variables measured in the screen.

(A) Heatmap of Pearson correlation coefficient matrix for variables used to generate PC plot in Figure 1D.

(B) Total variance explained by top PC components.

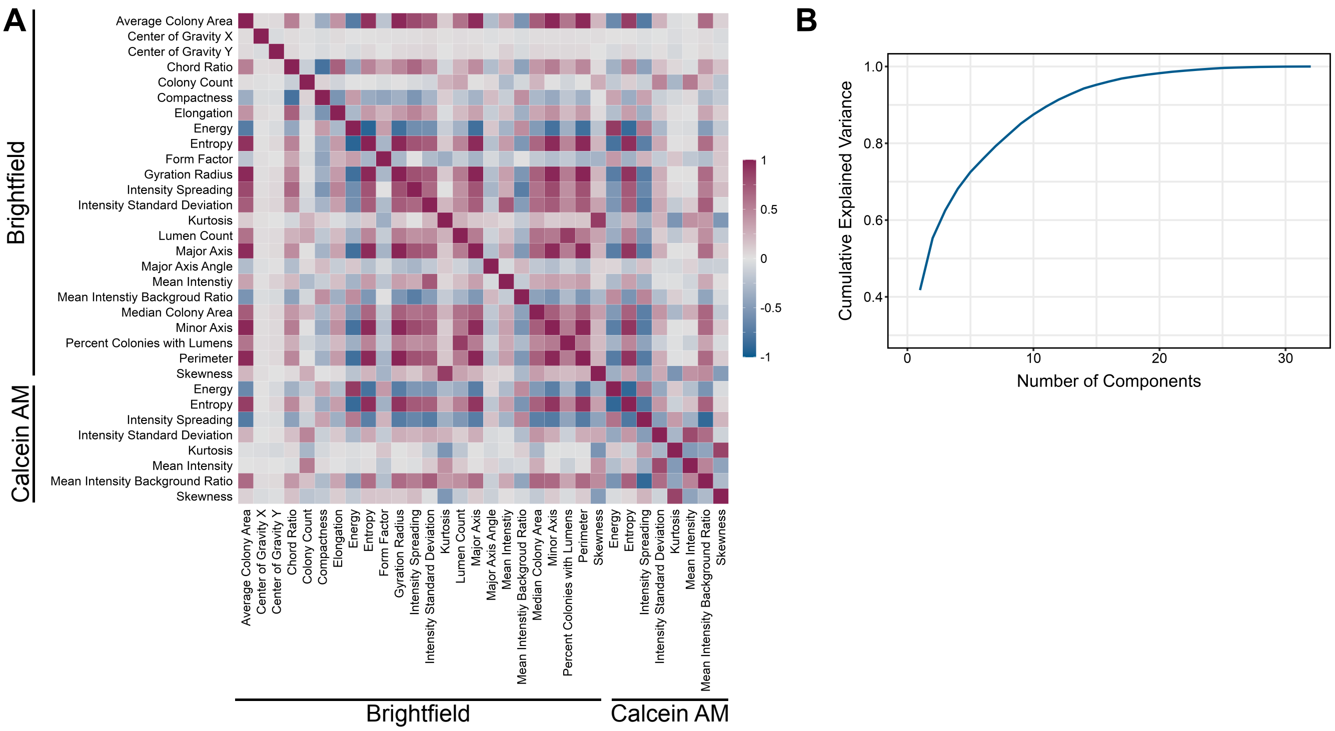

Supplemental Figure 2. Correlation among all the screening variables measured in the screen with P4G11 control.

(A) Heatmap of Pearson correlation coefficient matrix for variables used to generate PC plot in Figure 2A.

(B) Total variance explained by top PC components.

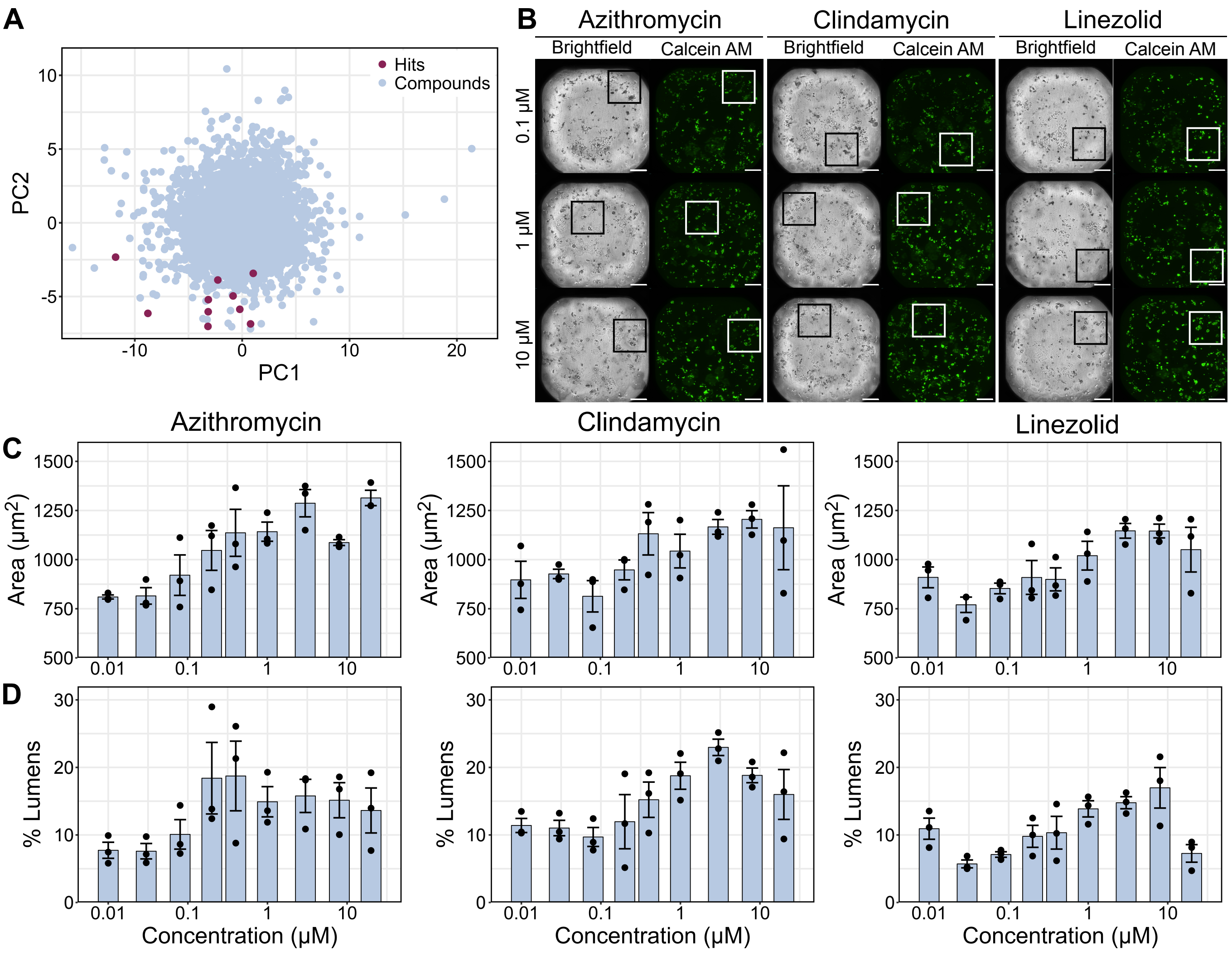

Supplemental Figure 3. Additional analysis of top three hits identified in screen.

(A) Principal component analysis (PCA) of morphological characteristics of colonies from high-throughput drug screen. Each dot represents a compound-treated well from the screen. Wells identified as hits in both median colony area and percent colonies with lumens are highlighted.

(B) Full well brightfield and fluorescent (Calcein AM) images for all three concentrations of the top three hits identified in the screen. Boxes indicate inset location for images used in Figure 3F. Images taken with ImageXpress confocal HT.ai automated high-content imaging system at 4x magnification. (Scale bars: 500 µm).

(C-D) Median colony area (C) and percent colonies with lumens (D) of wells over 9-point dose curve for top three hits in the screen. Cells plated as described for the screen. Points represent median from 3 technical replicates (wells) from experiment. Plotted as mean ±SEM.

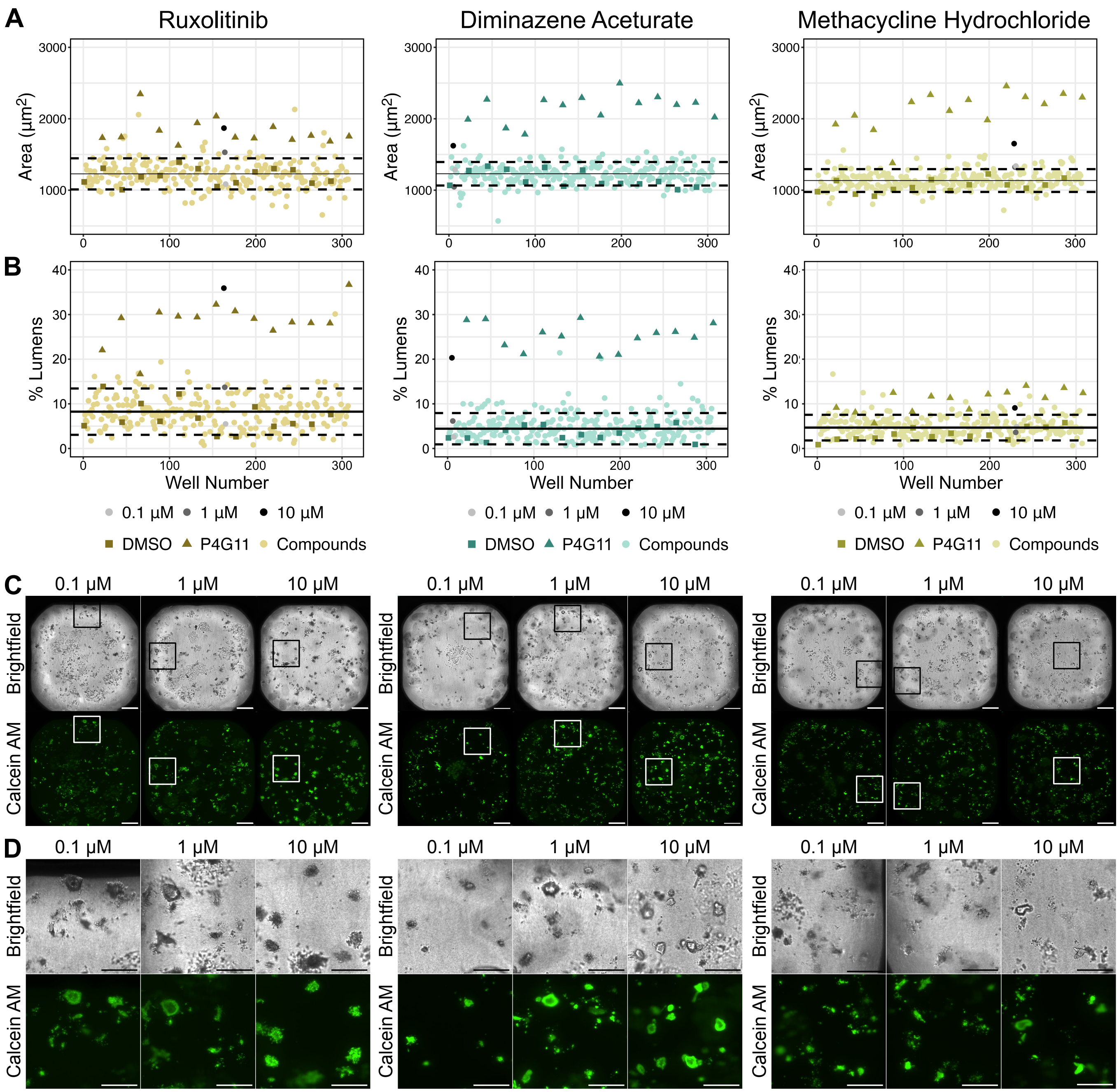

Supplemental Figure 4. Additional hits that re-epithelialize CRC colonies in 3D collagen.

(A-B) Comparison of (A) median colony area and (B) percent colonies for three additional hits (as indicated on top) tested at 0.1, 1, and 10 µM, compared to other wells from the same plate from screen treated with DMSO, P4G11, or other compounds. Well number was assigned based on order of the wells. Solid line indicates the median for all wells of the plate and the dotted lines denote the interquartile range.

(C) Brightfield and fluorescent (Calcein AM) confocal images of wells treated with additional hits from the screen. (Scale bars: 500 µm). Brightfield images are best in focus projection images of z-stack. Calcein AM images are

maximum intensity projection images of the z-stack. Boxes indicate inset location for images used in Figure S4D. Images taken with ImageXpress confocal HT.ai automated high-content imaging system at 4x magnification.

(D) Insets of brightfield and fluorescent (Calcein AM) confocal images shown in Figure S4C. (Scale bars: 250 µm).

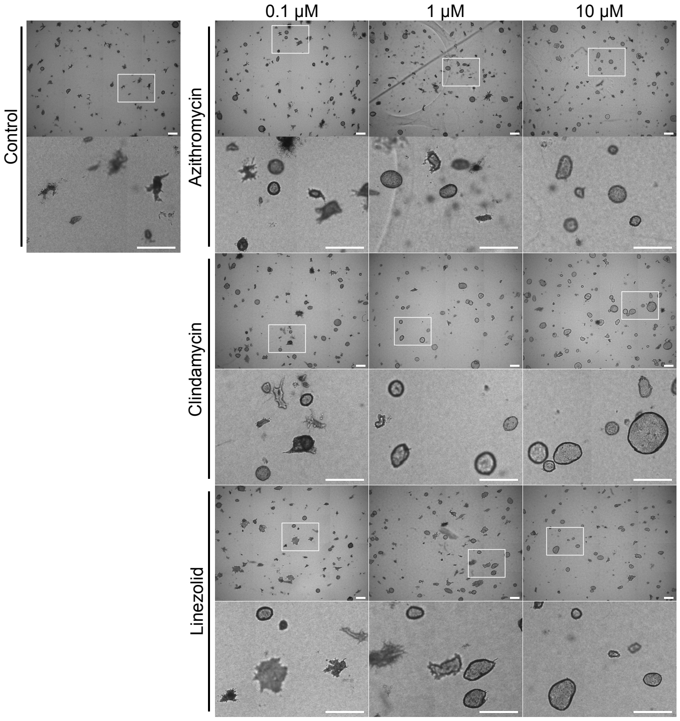

Supplemental Figure 5: Validation of re-circularization by top three hits from drug screen in 24-well 3D type I collagen cultures.

Representative whole well images and insets of SC cells grown in 24-well type I collagen cultures for 14 days with increasing concentrations of azithromycin, clindamycin, and linezolid. Images taken using MuviCyte Live-Cell Imaging System. Boxes indicate inset location (Scale bars: 500 μm, Insets: 500 μm).

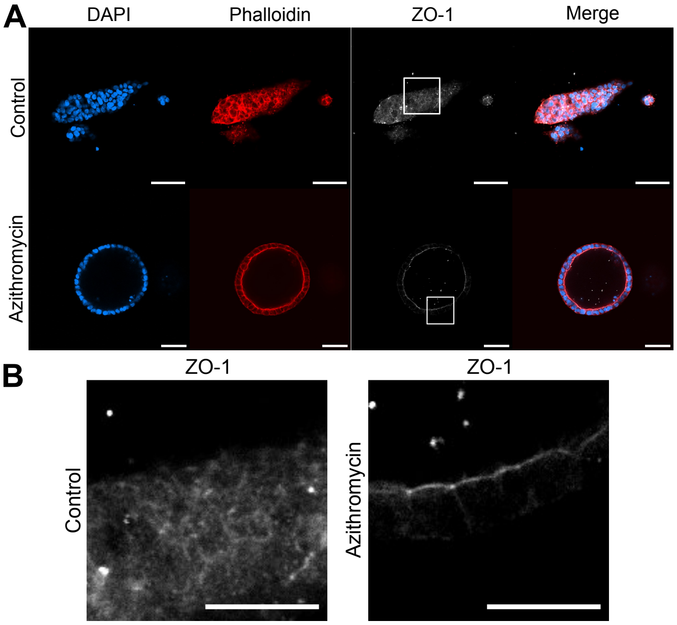

Supplemental Figure 6: Altered ZO-1 localization in SC colonies treated with azithromycin.

(A) Fluorescent confocal images of SC cell colonies treated with azithromycin. SC cells were seeded in type I collagen and incubated with 10 µM azithromycin for 14 days. Collagen layer containing colonies was isolated, fixed, and stained for DAPI (blue), Phalloidin (red), and ZO-1 (grey). Colonies were imaged through the equatorial plane using a 10x objective. (Scale bars: 100 µm). Boxes indicate inset location for images in Figure S6B.

(B) Magnified insets of E-cadherin from images shown in Figure S6A. (Scale bars: 50 µm).

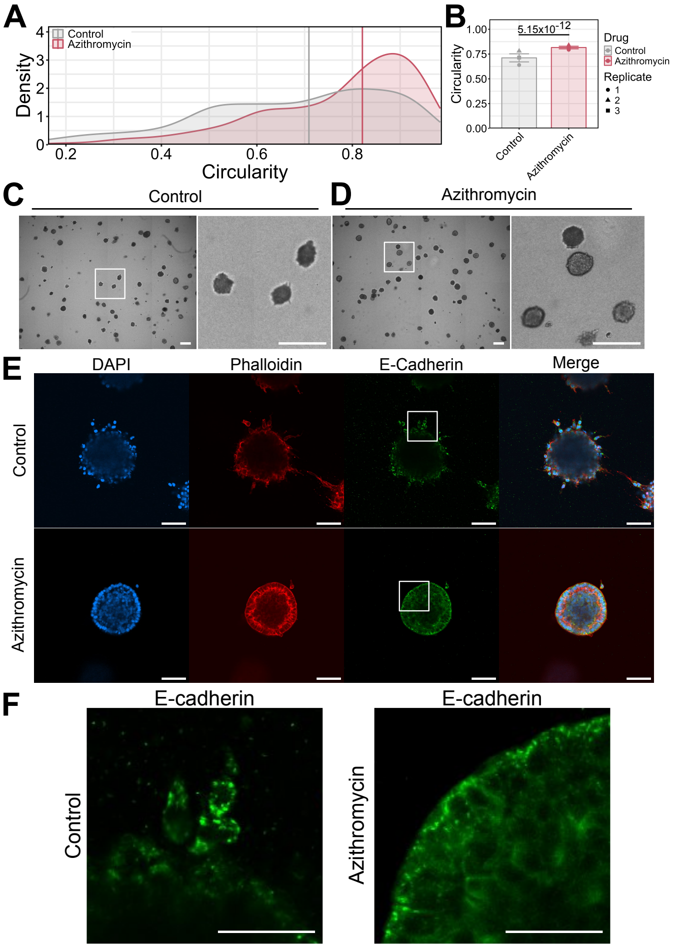

Supplemental Figure 7: Re-circularization of additional CRC cell line by azithromycin in 3D type I collagen cultures.

(A-B) Quantification of SW480 cell colony circularity when treated with 10 µM azithromycin. SW480 cells were seeded in type I collagen and incubated with azithromycin for 10 days. Results are quantified as circularity index. Higher circularity indicates rounder colonies and lower circularity index indicates spiky colonies. Density plot (A) of the circularity for all colonies from each treatment for all three biological replicates. Vertical line indicates the median circularity for each treatment condition. Bar graph (B) depicting median circularity for each biological replicate. n>150 for each biological replicate from 3 technical replicates. Plotted as mean±SEM. Wilcoxon rank-sum test, p-value indicated.

(C-D) Representative whole well images of SW480 cells grown in 24-well type I collagen cultures for 10 days without (C) and with 10 µM azithromycin (D). Images taken using MuviCyte Live-Cell Imaging System. Boxes indicate inset location. (Scale bars: 500 µm, Inset: 500 µm).

(E) Fluorescent confocal images of SW480 cell colonies treated with 10 µM azithromycin for 10 days in type I collagen. Collagen layer containing colonies was isolated, fixed, and stained for DAPI (blue), Phalloidin (red),

and E-cadherin (green). Colonies were imaged through the equatorial plane using a 10x objective. (Scale bars: 100 μm). Boxes indicate inset location for images in Figure S7F.

(F) Magnified insets of E-cadherin from images shown in Figure S7E. (Scale bars: 50 μm).

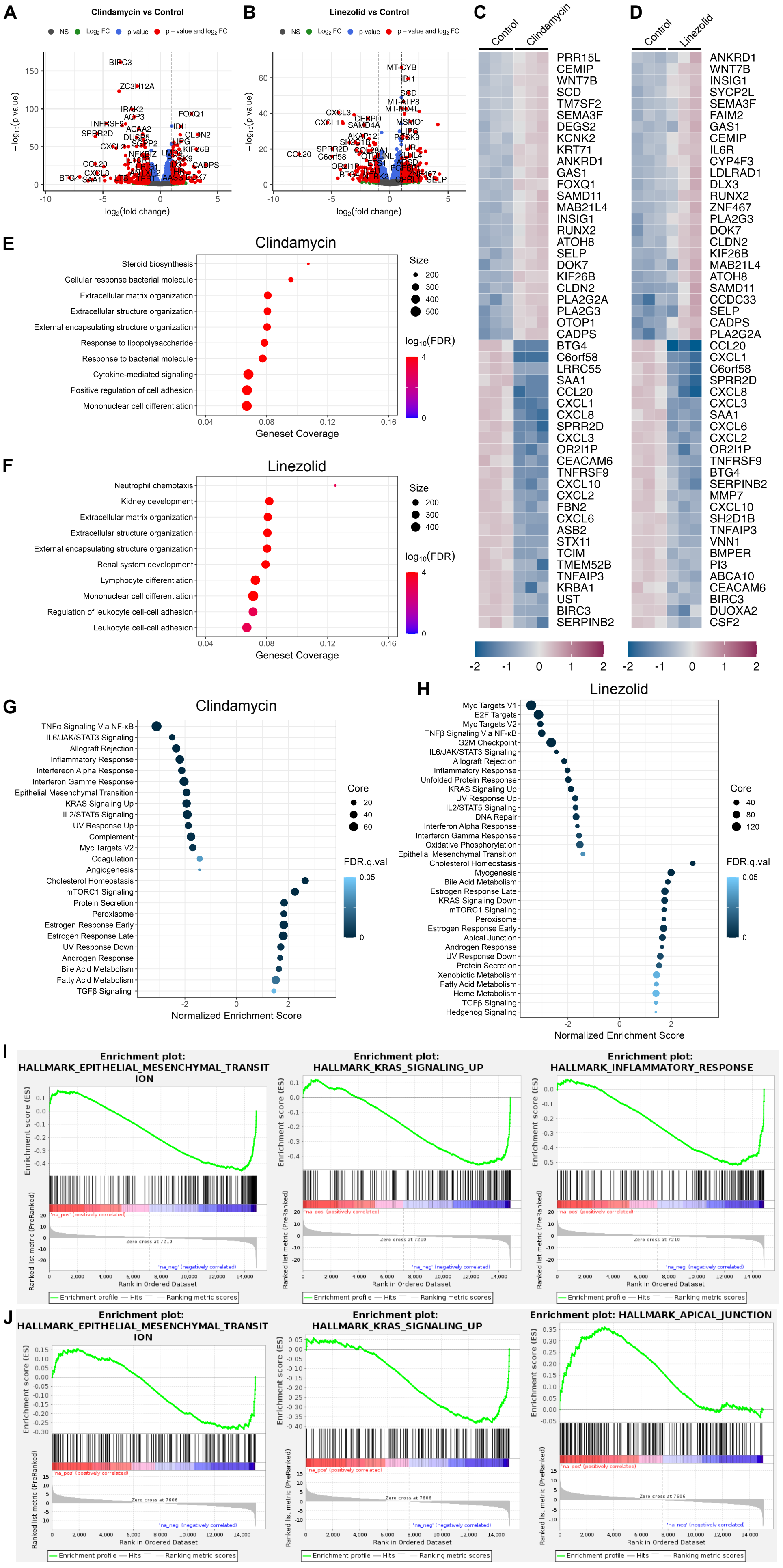

A
Clindamycin vs Control
NS
Log2 FC
p-value
p – value and log2 FC
– log10(p value)
log2(fold change)
BIRC3
ZC3H12A
IRAK2
AQP3
TNFRSF9
SPRR2D
FOXQ1
ID1
CLDN2
LIPG
KIF26B
CADPS
CCL20
CXCL8
CXCL3
BTG4
SAA1
B
Linezolid vs Control
MT-CYB
IDI1
SCD
MT-ATP8
MT-ND4L
MSMO1
CXCL3
CXCL1
CEBPD
SAMD4A
CCL20
SPRR2D
C6orf58
SELP
C
Control
Clindamycin
PRR15L
CEMIP
WNT7B
SCD
TM7SF2
SEMA3F
DEGS2
KCNK2
KRT71
ANKRD1
GAS1
FOXQ1
SAMD11
MAB21L4
INSIG1
RUNX2
ATOH8
SELP
DOK7
KIF26B
CLDN2
PLA2G2A
PLA2G3
OTOP1
CADPS
BTG4
C6orf58
LRRC55
SAA1
CCL20
CXCL1
CXCL8
SPRR2D
CXCL3
OR2I1P
CEACAM6
TNFRSF9
CXCL10
CXCL2
FBN2
CXCL6
ASB2
STX11
TCIM
TMEM52B
TNFAIP3
KRBA1
UST
BIRC3
SERPINB2
-2 -1 0 1 2
D
Control
Linezolid
ANKRD1
WNT7B
INSIG1
SYCP2L
SEMA3F
FAIM2
GAS1
CEMIP
IL6R
CYP4F3
LDLRAD1
DLX3
RUNX2
ZNF467
PLA2G3
DOK7
CLDN2
KIF26B
MAB21L4
ATOH8
SAMD11
CCDC33
SELP
CADPS
PLA2G2A
CCL20
CXCL1
C6orf58
SPRR2D
CXCL8
CXCL3
SAA1
CXCL6
CXCL2
OR2I1P
TNFRSF9
BTG4
SERPINB2
MMP7
CXCL10
SH2D1B
TNFAIP3
VNN1
BMPER
PI3
ABCA10
CEACAM6
BIRC3
DUOXA2
CSF2
-2 -1 0 1 2
E
Clindamycin
Steroid biosynthesis
Cellular response bacterial molecule
Extracellular matrix organization
Extracellular structure organization
External encapsulating structure organization
Response to lipopolysaccharide
Response to bacterial molecule
Cytokine-mediated signaling
Positive regulation of cell adhesion
Mononuclear cell differentiation
Size
200
300
400
500
log10(FDR)
Geneset Coverage
F
Linezolid
Neutrophil chemotaxis
Kidney development
Extracellular matrix organization
Extracellular structure organization
External encapsulating structure organization
Renal system development
Lymphocyte differentiation
Mononuclear cell differentiation
Regulation of leukocyte cell-cell adhesion
Leukocyte cell-cell adhesion
Size
200
300
400
log10(FDR)
Geneset Coverage
G
Clindamycin
TNFα Signaling Via NF-κB
IL6/JAK/STAT3 Signaling
Allograft Rejection
Inflammatory Response
Interfereon Alpha Response
Interferon Gamme Response
Epithelial Mesenchymal Transition
KRAS Signaling Up
IL2/STAT5 Signaling
UV Response Up
Complement
Myc Targets V2
Coagulation
Angiogenesis
Cholesterol Homeostasis
mTORC1 Signaling
Protein Secretion
Peroxisome
Estrogen Response Early
Estrogen Response Late
UV Response Down
Androgen Response
Bile Acid Metabolism
Fatty Acid Metabolism
TGFβ Signaling
Core
20
40
60
FDR.q.val
Normalized Enrichment Score
H
Linezolid
Myc Targets V1
E2F Targets
Myc Targets V2
TNFβ Signaling Via NF-κB
G2M Checkpoint
IL6/JAK/STAT3 Signaling
Allograft Rejection
Inflammatory Response
Unfolded Protein Response
KRAS Signaling Up
UV Response Up
IL2/STAT5 Signaling
DNA Repair
Interferon Alpha Response
Interferon Gamma Response
Oxidative Phosphorylation
Epithelial Mesenchymal Transition
Cholesterol Homeostasis
Myogenesis
Bile Acid Metabolism
Estrogen Response Late
KRAS Signaling Down
mTORC1 Signaling
Peroxisome
Estrogen Response Early
Apical Junction
Androgen Response
UV Response Down
Protein Secretion
Xenobiotic Metabolism
Fatty Acid Metabolism
Heme Metabolism
TGFβ Signaling
Hedgehog Signaling
Core
40
80
120
FDR.q.val
Normalized Enrichment Score
I
Enrichment plot: HALLMARK_EPITHELIAL_MESENCHYMAL_TRANSITION
Enrichment plot: HALLMARK_KRAS_SIGNALING_UP
Enrichment plot: HALLMARK_INFLAMMATORY_RESPONSE
Enrichment score (ES)
Ranked list metric (PreRanked)
'na_pos' (positively correlated)
Zero cross at 7210
'na_neg' (negatively correlated)
Rank in Ordered Dataset
Enrichment profile — Hits Ranking metric scores
J
Enrichment plot: HALLMARK_EPITHELIAL_MESENCHYMAL_TRANSITION
Enrichment plot: HALLMARK_KRAS_SIGNALING_UP
Enrichment plot: HALLMARK_APICAL_JUNCTION
Enrichment score (ES)
Ranked list metric (PreRanked)
'na_pos' (positively correlated)
Zero cross at 7606
'na_neg' (negatively correlated)
Rank in Ordered Dataset
Enrichment profile — Hits Ranking metric scores

Supplemental Figure 8. Transcriptional analysis of 3D CRC colonies treated with clindamycin or linezolid

(A-B) Volcano plot for all comparisons all correlation PCA for control vs clindamycin-treated (A) and control vs linezolid-treated (B) 3D cultures. Differential expression analysis criteria: absolute fold change ≥ 2 and FDR adjusted p value ≤ 0.05.

(C-D) Heatmap of top 50 differentially expressed transcripts in control vs clindamycin-treated (C) and control vs linezolid-treated (D) 3D cultures in triplicates; purple = up, blue = down (expression scale in inset).

(E-F) WebGestalt-based control vs clindamycin-treated (E) and control vs linezolid-treated (F) pathway over-representation analysis. Top 10 biological processes significantly overrepresented (FDR ≤ 0.5) are depicted with their respective enrichment ratios.

(G-H) Hallmark gene set enrichment analysis comparison of RNA-seq data from control vs clindamycin-treated (G) and control vs linezolid-treated (H) 3D cultures. Hallmark pathways are indicated on the left and are represented as bubbles on the Normalized enrichment score on x-axis. Bubble size indicates core enrichment or number of leading-edge genes and bubble color represents FDR (false discovery rate); scales of both are indicated on the right.

(I-J) Gene set enrichment analysis (GSEA) of RNA-seq data from control vs clindamycin-treated (I) and control vs linezolid-treated (J) cultures. Three select categories of interest are shown. Abbreviations: NES = Normalized enrichment score; FDR = False discovery rate q-values.

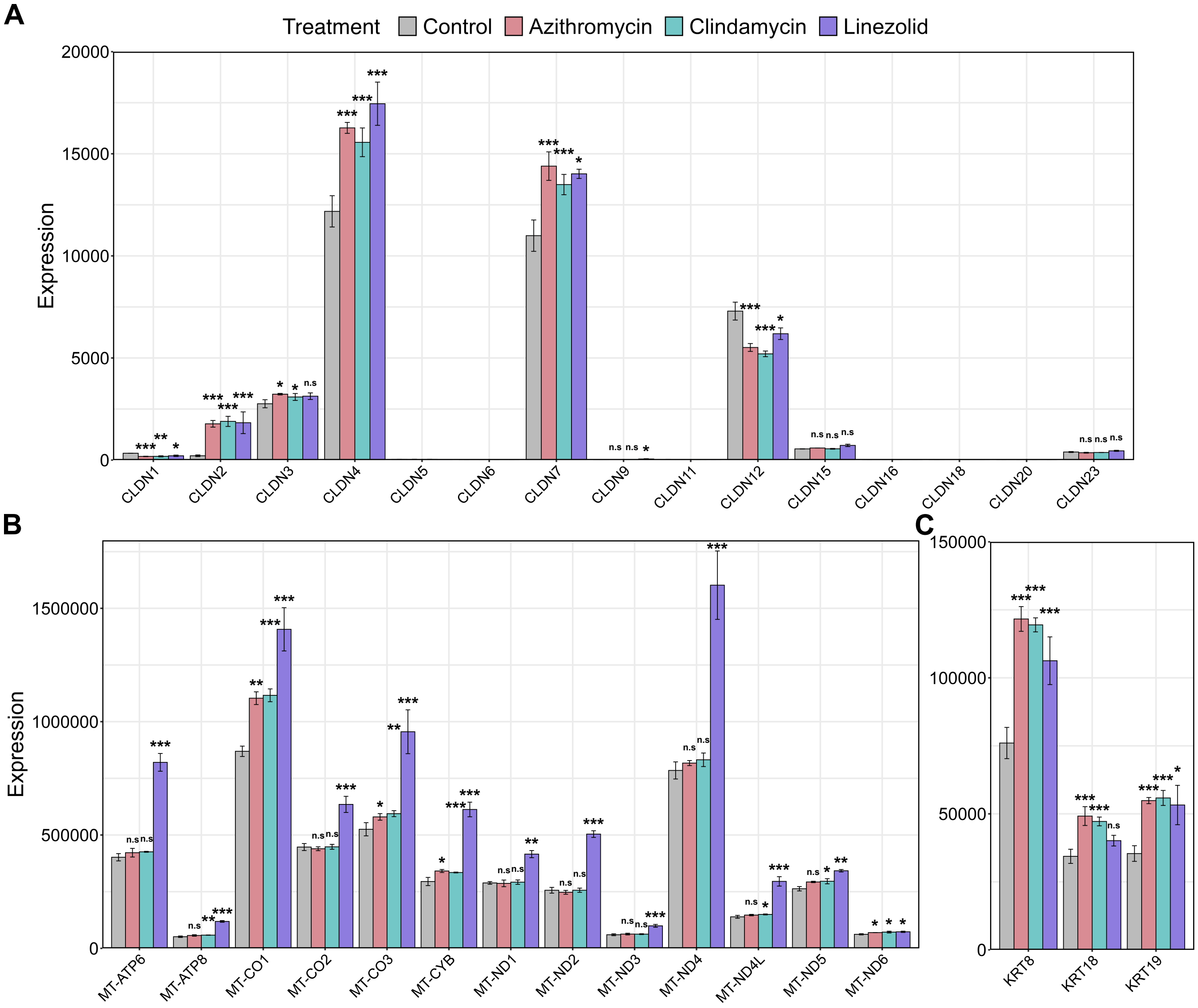

Supplemental Figure 9: RNA expression of select gene classes during antibiotic treatment.

(A-C) Comparison of RNA expression level (RNA-seq) of claudins (A), mitochondrial encoded proteins (B), and keratins (C) between control (untreated) and SC cell colonies grown in 3D collagen for 12 days in the presence of azithromycin, clindamycin, or linezolid. Plotted as mean±SEM. Statistical analysis: ns = not significant; * = p<0.05; ** = p<0.001; *** = p<0.0001 (FDR adjusted p-value).

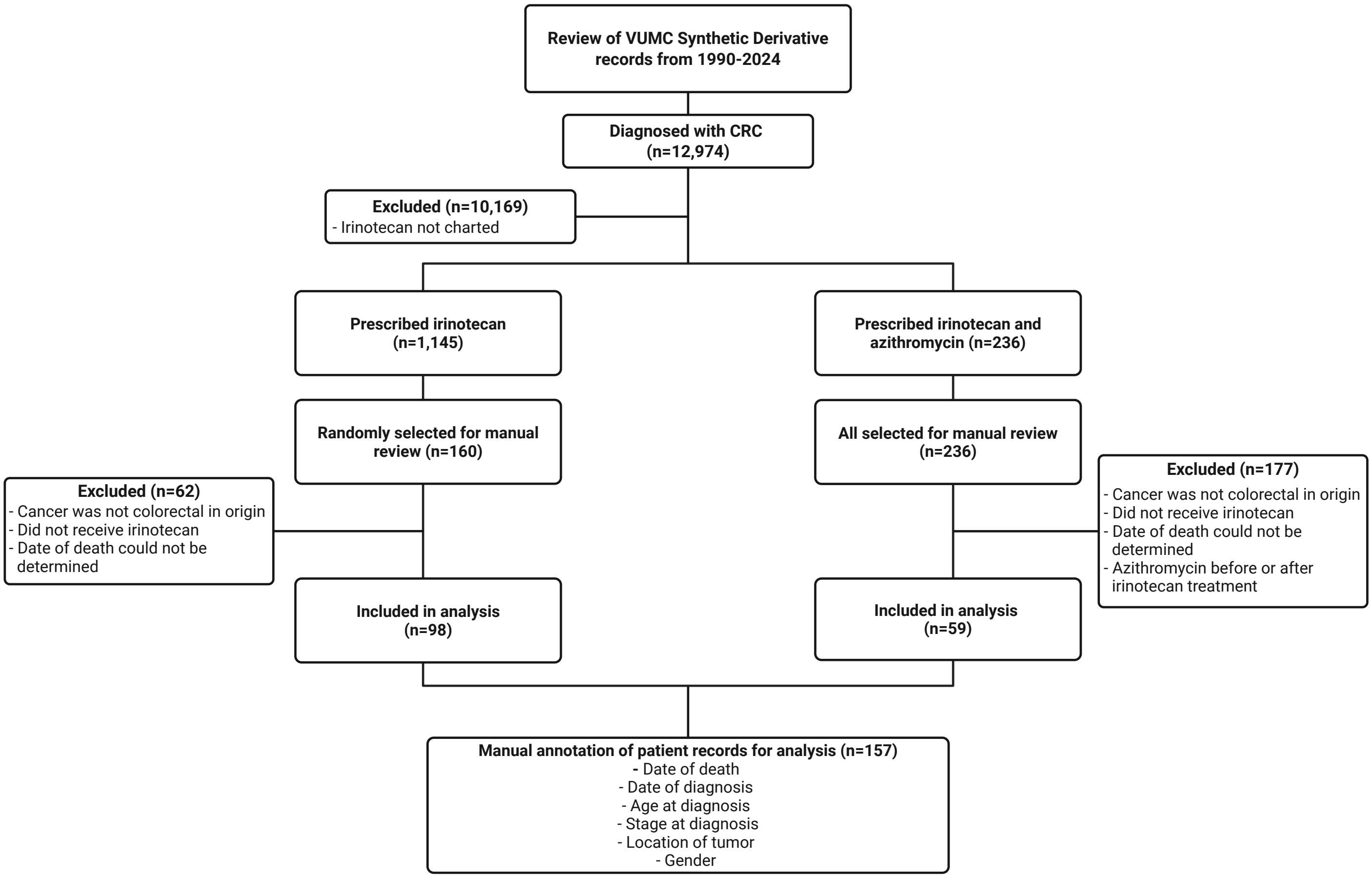

Supplemental Figure 10. Flow chart showing inclusion and exclusion of patients in retrospective study. Created in BioRender. Harmych, S. (2025) https://BioRender.com/v3gwd3w.

	No Azithromycin (n=98)	Azithromycin (n=59)	Total (n=157)	p-value
**Age at diagnosis (years)**				0.93
Non-missing	98	59	157	
Median (Q1-Q3)	53.5 (47.25, 62.0)	53.0 (46.5, 62.5)	53.0 (47.0, 62.0)	
**Age at diagnosis**				0.98
Non-missing	98	59	157	
Early Onset (< 50)	33 (34%)	20 (34%)	53 (34%)	
Late Onset (≥ 50)	65 (66%)	39 (66%)	104 (66%)	
**Gender**				0.76
Non-missing	98	59	157	
Male	54 (55%)	34 (58%)	88 (56%)	
Female	44 (45%)	25 (42%)	69 (44%)	
**Race**				0.15
Non-missing	94	58	152	
Caucasian	74 (79%)	51 (88%)	125 (82%)	
Other	20 (21%)	7 (12%)	27 (18%)	
**Tumor Stage**				015
Non-missing	98	59	157	
Early (Stage 1-3)	29 (30%)	24 (41%)	53 (34%)	
Late (Stage 4)	69 (70%)	35 (59%)	104 (66%)	
**Tumor Location**				0.81
Non-missing	90	58	148	
Left	46 (51%)	32 (55%)	78 (53%)	
Right	21 (23%)	11 (19%)	32 (22%)	
Rectum	23 (26%)	15 (26%)	38 (26%)	
**Survival Status**				0.03
Non-missing	98	59	157	
Alive	28 (29%)	27 (46%)	55 (35%)	
Dead	70 (71%)	32 (54%)	102 (65%)	
**Overall follow-up or survival time (days)**				<0.01
Non-missing	98	59	157	
Median (Q1, Q3)	672 (293, 1343)	1121 (788, 1826)	870 (442, 1630)	

Supplemental Table 1. Clinicopathological features of patients by azithromycin usage.

Characteristics are presented as medians with interquartile ranges or as frequencies with percentages. Comparisons between treatment groups were made using the Wilcoxon rank-sum test except for tumor location which was made using the chi-squared test. Age at diagnosis indicates age of the patient at initial diagnosis of cancer, which was broken down into early and late onset with early onset being defined at <50 years of age. Tumor stage indicates the stage of the tumor at initial diagnosis, not necessarily the stage of the cancer when irinotecan treatment was given. Patients with early-stage tumors at diagnosis were likely to have received irinotecan following disease progression or recurrence based on when irinotecan is commonly given. Overall follow-up or survival time indicates patient survival following initial diagnosis up to five years (1826 days).
