# Supplementary material for: 3D collagen high-throughput screen identifies drugs that induce epithelial polarity and enhance chemotherapy response in colorectal cancer
Source: Commun Biol. 2025 Aug 22;8:1261. doi: 10.1038/s42003-025-08699-0 (PMC12373842; doi:10.1038/s42003-025-08699-0)
Supplement: Supplementary file 3 — Description of Additional Supplementary Files [file 42003_2025_8699_MOESM3_ESM.pdf]

Description of additional supplementary fil

eFile name: Supplemental Data 1

Description: The source data behind the graphs in the paper including morphological measurements for all drugs tested in the high-throughput drug screen.
