# Supplementary material for: 3D collagen high-throughput screen identifies drugs that induce epithelial polarity and enhance chemotherapy response in colorectal cancer
Source: Commun Biol. 2025 Aug 22;8:1261. doi: 10.1038/s42003-025-08699-0 (PMC12373842; doi:10.1038/s42003-025-08699-0)
Supplement: Supplementary file 5 — NR Reporting Summary [file 42003_2025_8699_MOESM5_ESM.pdf]

nature portfolio

Corresponding author(s): Bhuminder Singh

Last updated by author(s): Jul 25, 2025

Reporting Summary

Nature Portfolio wishes to improve the reproducibility of the work that we publish. This form provides structure for consistency and transparency in reporting. For further information on Nature Portfolio policies, see our Editorial Policies and the Editorial Policy Checklist.

Statistics

For all statistical analyses, confirm that the following items are present in the figure legend, table legend, main text, or Methods section.

n/a	Confirmed	
☐	☒	The exact sample size (*n*) for each experimental group/condition, given as a discrete number and unit of measurement
☐	☒	A statement on whether measurements were taken from distinct samples or whether the same sample was measured repeatedly
☐	☒	The statistical test(s) used AND whether they are one- or two-sided<br>*Only common tests should be described solely by name; describe more complex techniques in the Methods section.*
☐	☒	A description of all covariates tested
☐	☒	A description of any assumptions or corrections, such as tests of normality and adjustment for multiple comparisons
☐	☒	A full description of the statistical parameters including central tendency (e.g. means) or other basic estimates (e.g. regression coefficient) AND variation (e.g. standard deviation) or associated estimates of uncertainty (e.g. confidence intervals)
☐	☒	For null hypothesis testing, the test statistic (e.g. *F*, *t*, *r*) with confidence intervals, effect sizes, degrees of freedom and *P* value noted<br>*Give P values as exact values whenever suitable.*
☒	☐	For Bayesian analysis, information on the choice of priors and Markov chain Monte Carlo settings
☐	☒	For hierarchical and complex designs, identification of the appropriate level for tests and full reporting of outcomes
☐	☒	Estimates of effect sizes (e.g. Cohen's *d*, Pearson's *r*), indicating how they were calculated

Our web collection on statistics for biologists contains articles on many of the points above.

Software and code

Policy information about availability of computer code

Data collection	Molecular devices software used for the collection of confocal images from screen. InCarta software from Molecular Devices used to collect phenotypic measurements of wells. MuviCyte software used for collection of 24-well images and MorphoLibJ plugin on FiJi was used to collect measurements.
Data analysis	Data analysis was conducted using R software and available open-source packages.

For manuscripts utilizing custom algorithms or software that are central to the research but not yet described in published literature, software must be made available to editors and reviewers. We strongly encourage code deposition in a community repository (e.g. GitHub). See the Nature Portfolio guidelines for submitting code & software for further information.

Data

Policy information about availability of data

All manuscripts must include a data availability statement. This statement should provide the following information, where applicable:

- Accession codes, unique identifiers, or web links for publicly available datasets
- A description of any restrictions on data availability
- For clinical datasets or third party data, please ensure that the statement adheres to our policy

Phenotypic measurements collected during the screen will be made available. Data collected from the Synthetic Derivative at VUMC cannot be made available at this time since it is patient data.

Research involving human participants, their data, or biological material

Policy information about studies with human participants or human data. See also policy information about sex, gender (identity/presentation), and sexual orientation and race, ethnicity and racism.

Reporting on sex and gender	Gender was controlled for as a confounding variable in survival studies conducted.
Reporting on race, ethnicity, or other socially relevant groupings	Race was controlled for as a confounding variable in survival studies conducted.
Population characteristics	Collected information such as: date of diagnosis, data of death, first recorded date of drug usage, age at diagnosis, location of colorectal cancer, gender, race, stage of cancer at diagnosis.
Recruitment	Patients were selected based on diagnosis with colorectal cancer in the last 5 years and the use of drugs of interest.
Ethics oversight	Vanderbilt IRB

Note that full information on the approval of the study protocol must also be provided in the manuscript.

Field-specific reporting

Please select the one below that is the best fit for your research. If you are not sure, read the appropriate sections before making your selection.

☒ Life sciences ☐ Behavioural & social sciences ☐ Ecological, evolutionary & environmental sciences

For a reference copy of the document with all sections, see nature.com/documents/nr-reporting-summary-flat.pdf

Life sciences study design

All studies must disclose on these points even when the disclosure is negative.

Sample size	Excluding studies done in 384-well format (screen and follow up dose curves) all experiments have three biological replicates.
Data exclusions	No data was excluded from analysis.
Replication	All experiments were replicated three times, excluding the screen and dose curves in 384-well format which could only be conducted once due to feasibility. Phenotype produced by hits in the screen were able to be replicated in larger format collagen cultures.
Randomization	Retrospective study of patient data could not be randomized.
Blinding	Blinding could not be conducted as analysis of patient data was done retrospectively. Hit identification during the screen was done blind. Names of drugs were not known until hit wells had been identified.

Reporting for specific materials, systems and methods

We require information from authors about some types of materials, experimental systems and methods used in many studies. Here, indicate whether each material, system or method listed is relevant to your study. If you are not sure if a list item applies to your research, read the appropriate section before selecting a response.

Materials & experimental systems

n/a	Involved in the study	
☐	☒	Antibodies
☐	☒	Eukaryotic cell lines
☒	☐	Palaeontology and archaeology
☒	☐	Animals and other organisms
☒	☐	Clinical data
☒	☐	Dual use research of concern
☒	☐	Plants

Methods

n/a	Involved in the study	
☒	☐	ChIP-seq
☒	☐	Flow cytometry
☒	☐	MRI-based neuroimaging

Antibodies

Antibodies used	Anti-E-cadherin (BD Transduction Laboratories #610181 mouse antibody), Anti-Integrin β1 Antibody, clone P4G11 was purchased

Antibodies used	from Sigma-Aldrich (Damstadt, Germany, #MAB1951). Anti-ZO-1 antibody was purchased from Invitrogen (Waltham, MA, USA, #61-7300).
Validation	Past studies in our lab have confirmed the re-epithithelialization effect of P4G11 (PMID: 28356422, 34037774).

Eukaryotic cell lines

Policy information about cell lines and Sex and Gender in Research

Cell line source(s)	SC1 cells. Generated by the Coffey laboratory and extensively characterized (PMID: 28320945, 24691442).
Authentication	Cells lines were generated in house and have been extensively characterized (see above).
Mycoplasma contamination	Cell line was tested for mycoplasma contamination at time of generation.
Commonly misidentified lines (See ICLAC register)	No commonly misidentified lines were used for this study.

Plants

Seed stocks	N/A
Novel plant genotypes	N/A
Authentication	N/A
